# Seizures induced by micturition: A rare form of reflex epilepsy

**DOI:** 10.1016/j.ebr.2021.100460

**Published:** 2021-05-31

**Authors:** Sara Casciato, Pier Paolo Quarato, Addolorata Mascia, Alfredo D'Aniello, Liliana G. Grammaldo, Giancarlo Di Gennaro

**Affiliations:** IRCCS Neuromed, Pozzilli (IS), Italy

## Abstract

• Micturition-induced seizures are a rare form of reflex epilepsy.
• Video-EEG monitoring is crucial for diagnosis.
• Symptomatogenic zone involves mesial fronto-parietal cortex.

Dear Editor,

Seizures induced by micturition are rarely reported in literature [1], [2], [3], [4], [5]. They may be difficult to distinguish from micturition-induced syncope, especially in cases in which loss of consciousness occurs early. We report a case of an 18-year-old left‐hand dominant boy with video-EEG documented spontaneous and micturition-induced seizures. He was admitted to our Epilepsy Monitoring Unit for recent onset of daily, paroxysmal episodes occurring at the end of urination, lasting for a few seconds. This was characterized by a painful sensation in his penis with an episode that included graspoing his genitals, rhythmical movements of the trunk with generalized stiffening, and involving preserved awareness. This condition resulted in feelings of fear to urinate with voluntional urinary retention and considerable psychological discomfort. The boy complained the occurrence of similar episodes also spontaneously occurred during sleep.

He was born from non-consanguineous parents by normal delivery after a normal pregnancy. No risk factors for epilepsy had been reported. Psychomotor developmental milestones were reached and were normal. No genitourinary or sexual dysfunctions had been reported.

General and neurological examinations were unremarkable. In particular, the external genitals appeared morphologically normal.

In addition, an outpatient urological evaluation, including ultrasonography of the lower urinary tract, performed a few days before the admission revealed no abnormalities.

High-field 3 T MRI brain scan was normal. The boy underwent long term video-EEG monitoring, that showed rare interictal spiking over the left fronto-temporal head region and vertex, exclusively during NREM (N1-N2) sleep. Moreover, video-EEG clearly demonstrated that micturition quickly precipitated stereotyped ictal events: nine reflex seizures were induced by micturition and one reflex focal to bilateral tonic-clonic seizure, as well as eleven focal spontaneous seizure were recorded.

Ictal clinical semiology of focal seizures consisted of an aura characterized by sudden genital pain followed by manipulation lasting few seconds, without impaired awareness. In the recorded focal to bilateral tonic-clonic seizure behavioural arrest, right head deviation and arm/truncal asymmetric tonic posturing (right > left) was also observed.

Ictal EEG was characterized by the onset of rhythmical delta activity evolving to sharp waves located over the midline invovling the central-parietal and left fronto-temporal channels, with the recruiting rhythm evolving to a bilateral tonic-clonic seizure (Fig. 1).

**Fig. 1:**
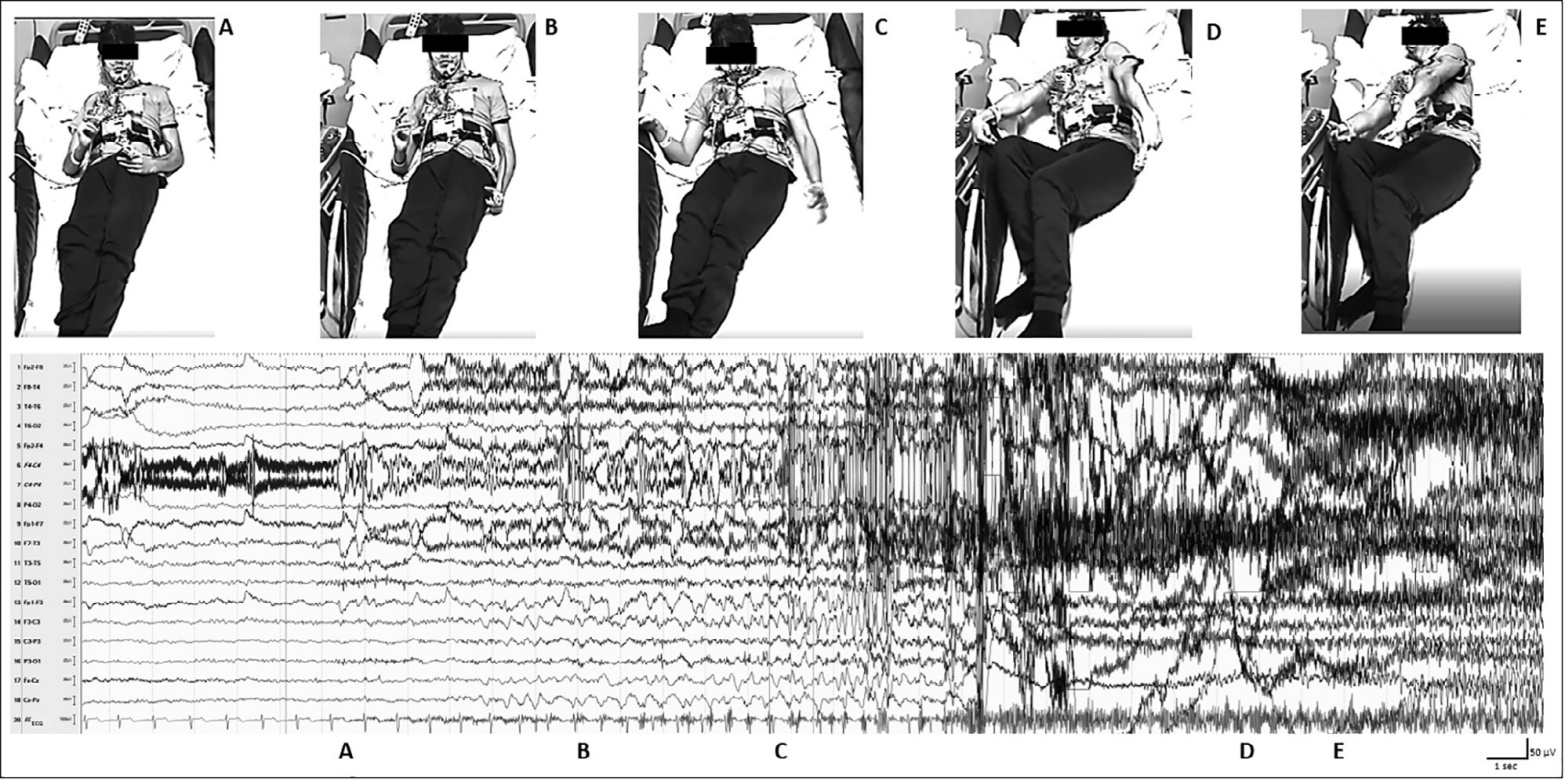
Video-EEG findings in the focal to bilateral tonic-clonic seizure: A-B: the subject while experiences the habitual aura characterized by sudden genital pain followed by manipulation; C: behavioral arrest and right grasping; D: right head deviation and arms/trunk asymmetric tonic posturing (right > left); E: evolution to bilateral tonic-clonic seizure. Ictal EEG disclosed rhythmical delta activity evolving in recruiting sharp waves, mainly located over midline central-parietal and left fronto-temporal channels, spreading over contralateral regions intermixed with muscular artifacts.

Based on history and electro-clinical data, we diagnosed a focal epilepsy of unknown etiology with micturition-induced reflex seizures. The boy was started on carbamazepine, that was promptly discontinued after a few days due to an allergic skin rash. He was then switched to lacosamide, after which he remained seizure free for 3 months with a normal follow-up standard EEG.

Reflex seizures triggered by micturition are very rare, and few cases have been previously reported [1], [2], [3], [4], [5]. They may occur as an isolated entity or as a part of a focal or generalized epilepsy syndrome.

We report a case with both focal micturition-induced and spontaneous seizures, as previously described in other reflex epilepsy reports [1], [3]. Ictal EEG features showed an involvement of the midline central-parietal and left fronto-temporal regions. Accordingly, ictal clinical findings suggest that the epileptogenic network reliably include the left medial aspects of parietal lobe, with anterior spreading towards mesial frontal cortex.

Micturition is a complex physiological process that involves different hierarchically integrated anatomical central and peripheral structures, including cerebral cortex – in its mesial frontal, anterior cingulate and insular aspects -, brainstem and spinal cord [6]. Although the exact pathophysiological mechanism has not been fully elucidated, ictal EEG and single photon emission computed tomography findings suggest a central role of the mesial frontal cortex [7]. In another study, interictal magnetoencephalography showed that the activation cluster, corresponded to the ictal EEG onset, was localized in the left midline parietal region suggesting that this region may be closely networked with the frontal micturition center [1].

To our knowledge, only ten cases with micturition reflex epilepsy have been reported so far.

On the one hand, most of previous cases had similarities to the case we are reporting: unknown etiology, a midline-central epileptogenic zone, tonic seizures, and normal urinary tract function. On the other hand, our patient experienced not only reflex but also spontaneous seizures and did not show co-existing neurodevelopmental delay as often reported [1], [6].

In conclusion, we reported this case to further describe the anatomic-electroclinical correlation of the epileptogenic networks’ hypothesis of this extremely uncommon epilepsy syndrome that is often of unknown etiology. We also aimed to emphasize the crucial role of video-EEG monitoring in the correct differential diagnosis of rarely encountered paroxysmal events, that must be differentiated from more‐common conditions including micturition syncope, urinary incontinence due to sphincter relaxation during generalized seizures, and the ictal urge to urinate in patients wtih nondominant temporal lobe epilepsy [8], [9].

## Financial disclosures

This work did not receive any specific grant from funding agencies in the public, commercial, or not-for-profit sectors.

## Declaration of Competing Interest

The authors declare that they have no known competing financial interests or personal relationships that could have appeared to influence the work reported in this paper.
